# Regulation of Lysozyme Activity by Human Hormones

**DOI:** 10.52547/ibj.3614

**Published:** 2022-03-15

**Authors:** Timofei A. Pankratov, Andrey V. Gannesen, Yuri A. Nikolaev

**Affiliations:** S.N.Winogradsky Institute of Microbiology, Research Center of Biotechnology of the Russian Academy of Sciences, Moscow 119071, Russia

**Keywords:** Catecholamines, Estradiol, Muramidase, Natriuretic peptides

## Abstract

**Background:**

Lysozyme is a part of human and animal noncellular immunity. The regulation of its activity by hormones is poorly studied. The aim of this study was to test the *in vitro* activity of lysozyme in the presence of catecholamines, natriuretic hormones, and E2.

**Methods:**

Hormones were incubated with lysozyme, and the activity of lysozome was further determined using a test culture of *Micrococcus luteus* in the early exponential growth stage. The activity of lysozyme was assessed based on the rate of change in the OD of the test culture. Molecular docking was performed using SwissDock server (http://www.swissdock.ch/docking), and molecular structures were further analyzed and visualized in the UCSF Chimera 1.15rc software.

**Results:**

According to the results, EPN and NE increased lysozyme activity up to 180% compared to the hormone-free enzyme. Changing the pH of the medium from 6.3 to 5.5, increased the lysozyme activity in the presence of E2 up to 150-200 %. The results also showed that exposure to hormones could modify lysozyme activity, and this effect depends on the temperature and pH value. The molecular docking revealed a decrease in the activation energy of the active site of enzyme during the interaction of catecholamines with the amino acid residues, asp52 and glu35 of the active site.

**Conclusion:**

Our findings demonstrate an additional mechanism for the involvement of lysozyme in humoral regulation of nonspecific immunity with respect to human pathogenic microflora and bacterial skin commensals by direct modulation of its activity using human hormones.

## INTRODUCTION

Lysozyme (N-acetylmuramide glycanohydrolase, EC 3.2.1.17) is an ubiquitous enzyme present in all living organisms and induces noncellular immunity in birds and mammals. This enzyme is a component of lacrimal fluid and found in human milk, saliva, and other body fluids. It has also been identified in granulocytes, cells of the mononuclear phagocytic system, various exocrine glands, and articular cartilage^[^^1^^]^. The concentration of lysozyme in human blood plasma ranges from 7 to 13 mg/L^[^^2^^]^. 

There is scant information on the presence, characteristics, and regulation of the activity of lysozyme on skin surface. One of the first mentions of lysozyme activity on the skin refers to the work of Ogawa and coauthors^[^^3^^]^. Klenha and Krs^[^^4^^]^ have shown the presence of lysozyme on human’s skin surface at the amount of 5 × 10^-3^ µg/cm^2^. Papini et al.^[^^5^^]^ have demonstrated a consistent decrease in lysozyme activity in healthy skin in the untreated diabetic patients. They also found that the presence of lichen planus, psoriasis, conglobate acne, and chronic tuberculoid cutaneous toxoplasmosis did not affect serum and skin lysozyme levels in either normoglycemic or diabetic patients.

Lysozyme is effective against most Gram-negative bacteria in which lysis occurs even at low concentrations. At the same time, some Gram-positive bacteria inactivates lysozyme function through different mechanisms^[^^6^^,^^7^^]^, including O-acetylation. The most common human skin commensals or opportunistic pathogens among Gram-positive bacteria are members of the genera *Staphylococcus*, *Kytococcus*, *Cutibacterium*, and* Micrococcus*. Inside the cavities of skin glands and hair follicles, they are capable of forming stable biofilms that may provoke a number of diseases, such as vulgar acne, dermatitis, and folliculitis. At present, interest in the bacterial biofilms associating with human tissues has increased due to the influence of human’s hormonal regulation factors on their formation and activity. In particular, the systems of catecholamines (EPN and NE), biogenic peptides (natriuretic peptides), and reproductive hormones (E2 and androgen) in terms of their influence on the formation of bacterial biofilms and bacterial persistence have been widely studied *in vitro*^[^^8^^-^^10^^]^. 

Despite widespread studies in the effect of various regulators of lysozyme activity, including alkyl-hydroxybenzenes^[^^11^^]^, there are few data on the influence of human hormones on this enzyme. In this work, we tested the hypothesis of the influence of human hormones (EPN, NE, estradiol, ANP, and BNPs) on the activity of lysozyme from chicken egg white.

## MATERIALS AND METHODS

Lysozyme (20,000 E; Applichem, Germany) was dissolved in 0.07 M of phosphate buffer (pH 6.3). To assess the activity of the enzyme, we used a culture mediumcontaining *Micrococcus luteus* as a substrate^[12]^. We also evaluated the effect of hormones on lysozyme activity by incubating the enzyme solution with the hormones EPN, NE, E2, ANP, and BNP at concentrations (10-fold, 50-fold, and 100-fold) and close to the physiological norm in human blood plasma (Table 1). The mixture of aforesaid hormones with lysozyme was incubated in 2-ml eppendorf tubes at 34°, 37°, or 40 °C for 2 h.

*M. luteus* culture was prepared by two steps. The first step was performed to obtain an overnight culture of the stationary growth phase. For this purpose, one loop of the 24-h culture grown on agar was used to inoculate into 20 mL of the modified RCM medium (yeast extract, 10 g; peptone, 10 g; glucose, 5 g; sodium chloride, 5 g; sodium acetate, 3 g; deionized water, 1 L). The culture was then incubated in 50-mL conical flasks closed with cotton plugs with constant stirring at 180 rpm at 34°C. In the second step, 1 mL of the overnight culture was seeded into 20 mL of the fresh liquid RCM and incubated at the same above conditions for 3 h to reach an OD of 0.7-0.9. Then 20 μL of the enzyme solution, following incubation with the hormones (EPN, NE, E2, ANP, and BNP), was added to 2 mL of the bacterial suspension containing lysozyme (the final concentration of 400 μg/mL). The OD of the suspension was measured at 540 nm at 10-s intervals for 1 min using a spectrophotometer (JenWay 7315, Germany). The enzyme activity was expressed at the lysozyme concentration of 400 μg/mL and initial ODs (0.7-0.9) of *M. luteus* suspension in pH 6.3. To assess the effect of hormones on the degradation of *M. luteus* cells by lysozyme, hormone solutions together with *M. luteus* inoculum, were added to a liquid RCM at appropriate concentrations (Table 1). Cells from the overnight cultures were collected by centrifugation, the supernatant containing the hormones was discarded, and the pellet was resuspended in a fresh RCM and treated with lysozyme as described above; i.e., 20 µL of lysozyme solution was added to a 2-mL cell suspension with an OD of 0.7-0.9, and then the change in the OD was determined.

**Table 1:** Concentrations of hormones used in the study

**Hormone**	**Concentration (M)**			
	**Physiological norm**	**10×**	**50×**	**100×**
EPN	4.91 × 10^-12^	4.91 × 10^-11^	24.55 × 10^-11^	4.91 × 10^-10^
NE	3.55 × 10^-12^	3.55 × 10^-11^	17.75 × 10^-11^	3.55 × 10^-10^
E2	2.20 × 10^-13^	2.20 × 10^-12^	11.00 × 10^-12^	2.20 × 10^-11^
ANP	6.49 × 10^-15^	6.49 × 10^-14^	32.45 × 10^-14^	6.49 × 10^-13^
BNP	7.22 × 10^-15^	7.22 × 10^-14^	36.1 × 10^-14^	7.22 × 10^-13^

**Statistical analysis**

The data were analyzed by linear or exponential approximation using the MS Excel 2010 application package. The rate of change in OD was expressed in the units of OD/min by the equation coefficient of linear or exponential function. The coefficient in the approximation equation expressed the value of the change in OD as a function of exposure time. To obtain reliable data, each experiment was performed five to seven times. Differences (more than 12%) between control and experimental averages were considered significant and valid. Molecular docking was performed using SwissDock server (http://www. swissdock.ch/docking), and further analysis and visualization of the molecular structures of lysozyme were conducted in the UCSF Chimera 1.15rc software^[^^13^^]^.

## RESULTS

Preliminary experiments in which *M. luteus* cultures were incubated with the hormones showed that long-term incubation did not affect the properties of *M. luteus *cell wall, therefore, lysozyme activity did not change compared to the control (untreated cells). For this reason, all subsequent experiments were performed according to the procedure described in the "Materials and Methods" section. Since this work was carried out with a bacterium inhabiting human skin (skin surface temperature of 30.5-35.5 °C), incubation of lysozyme solution with hormones at 34 °C and pH of phosphate buffer 6.2-6.3 was considered as the standard conditions. Lysis rate values ranged from 0.14 to 0.3 units (OD/min) under standard conditions without hormone modification of lysozyme.

**Fig. 1:**
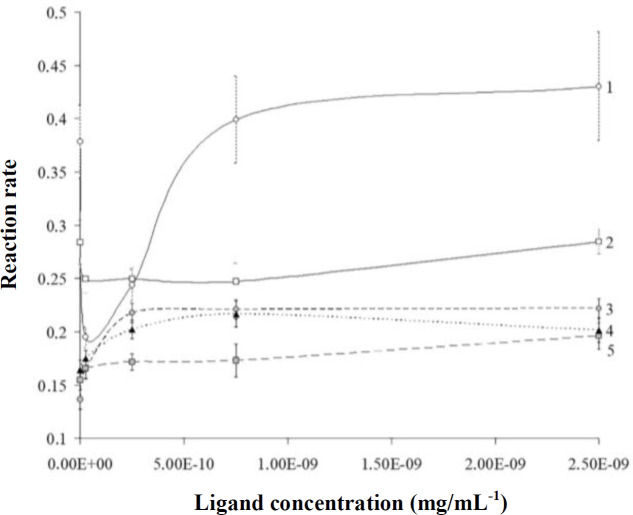
Dependence of lysozyme activity on ligand (hormone) concentration under the standard conditions (34 °C; pH 6.3), when treated with different concentrations of hormones. 1, NE; 2, BNP; 3, EPN; 4, E2; 5, ANP. Activity units -OD/min

Two types of effects relating to hormones on lysozyme activity can be distinguished in Figure 1. The first type includes EPN-, ANP-, and E2-activated lysozyme. A positive moderate correlation was revealed between lysozyme activity and EPN concentration, with correlation coefficient value of 0.61. For EPN, the maximum increase in activity reached 80% of the control, while for ANP, it was slightly lower. EPN had a weak stimulating effect on lysozyme activity, and the correlation coefficient for the ligand concentration and the rate of lytic activity of the enzyme in this case was close to 0.5. Maximum stimulation observed at the concentration of 50-folds, which was higher than that of the physiological concentration. This influence did not change with incremental concentrations. The second type of hormone, NE, decreased the activity of lysozyme by 30% at physiological concentration. However, when the concentration increased to 10 folds, the physiological concentration of the enzyme enhanced and reached a maximum of 3.55·10^-10^ mg/mL. The value of the correlation coefficient in this condition was 0.64, which was very close to the values found for EPN. The effect of BNP at all concentrations was weak and insignificant. Thus, following concentration increment from physiological norm to more than 10-fold of the physiological concentration, a trigger effect was not observed, which may be related to the saturation of hormone-binding cites with ligand molecules, leading to the lack of further change in the enzyme activity.

**Fig. 2:**
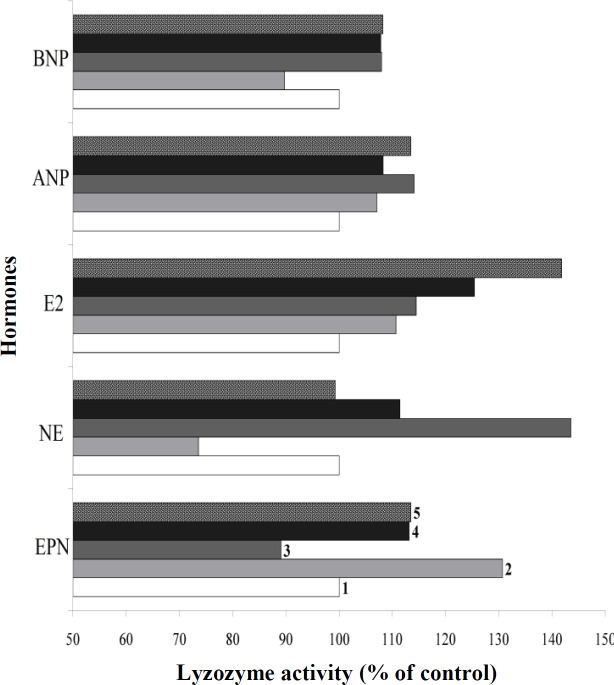
Changes in lysozyme activity values compared with the control when treated with physiological concentrations of hormones. 1, control (without hormones); 2, 34 °C; 3, 37 °C; 4, 40 °C; 5, pH 5.5

Lysozyme activity significantly decreased at 37 °C and was less noticeable at 40 °C at the physiological concentrations. The activity of E2, a steroid hormone, increased proportional to temperature, likely due to an alteration in the position of the molecule in the active site of lysozyme. The activity of the enzyme was not correlated with temperature when exposed to catecholamines. At 40 °C, the change in the lysozome activity was not fundamental for EPN/NE/NUP, except for weakening of the heating effect (Fig. 2). The impact of pH values on lysozyme activity in the presence of hormones was evaluated at 34 °C. At pH value of 7.5, we observed weak changes in lysozyme activity, within 5-7% of the control values (data not shown). The physiological concentrations of hormones increased the lysozyme activity at a pH of 5.5, which was comparable with that of at 40 °C and pH 6.2. The effect of pH value reduction on enzyme activity was significant when the lysozyme was treated with ES; the activity of the enzyme increased by 40-45% (Fig. 2).

A decrease in pH value to 5.5 affected the effectiveness of hormones on lysozyme activity in different ways. The level of enzyme activation decreased in the presence of ANP, and its inactivation occurred in the presence of EPN. No changes in the enzyme activity were observed in the presence of BNP, and no activity was detected upon NE incubation (Table 2). The activity of the enzyme treated with EPN increased to a greater extent than that under the standard conditions, with the maximum elevation at the highest concentration of the hormone.

**Fig. 3:**
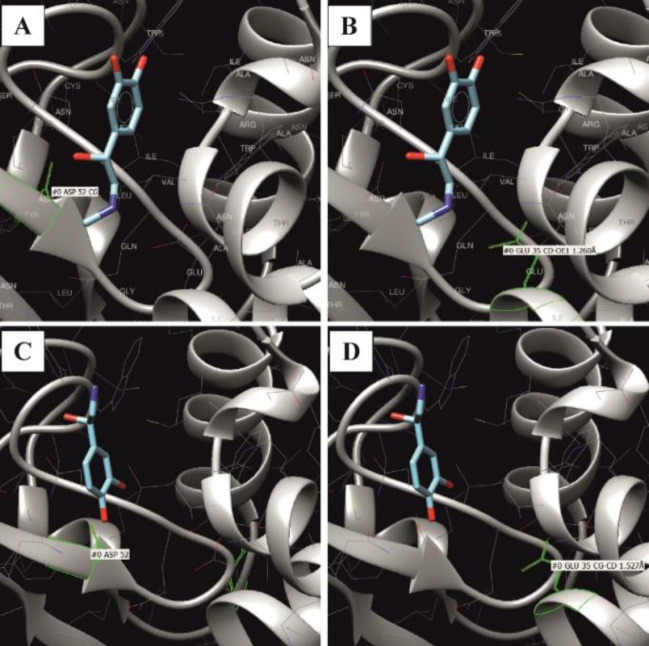
Position of the ligand: EPN (A, B) and NE (C, D) at the lowest Gibbs energy value in the lysozyme molecule. A is the position of asp52 residue, and B is the position of glu35 relative to the epinephrine molecule. C and D are the same for NE. The asp52 and glu35 residues are highlighted in green color

The underlying mechanism of hormone binding to lysozyme was evaluated to explain the observed effects. Modeling the interaction of EPN and NE with lysozyme (1 dpx, Protein Data Bank) showed that these two catecholamines took different energetically positions in the lysozyme molecule; minimum binding energy ∆G was -9.1077 for EPN and -7.2916 for NE. In the lysozyme molecule, both catecholamines occupied a location close to the ionized asparagine residue at position 52, which is one of the two critical centers of catalysis, along with glutamic acid at position 35 (Fig. 3).

**Fig. 4:**
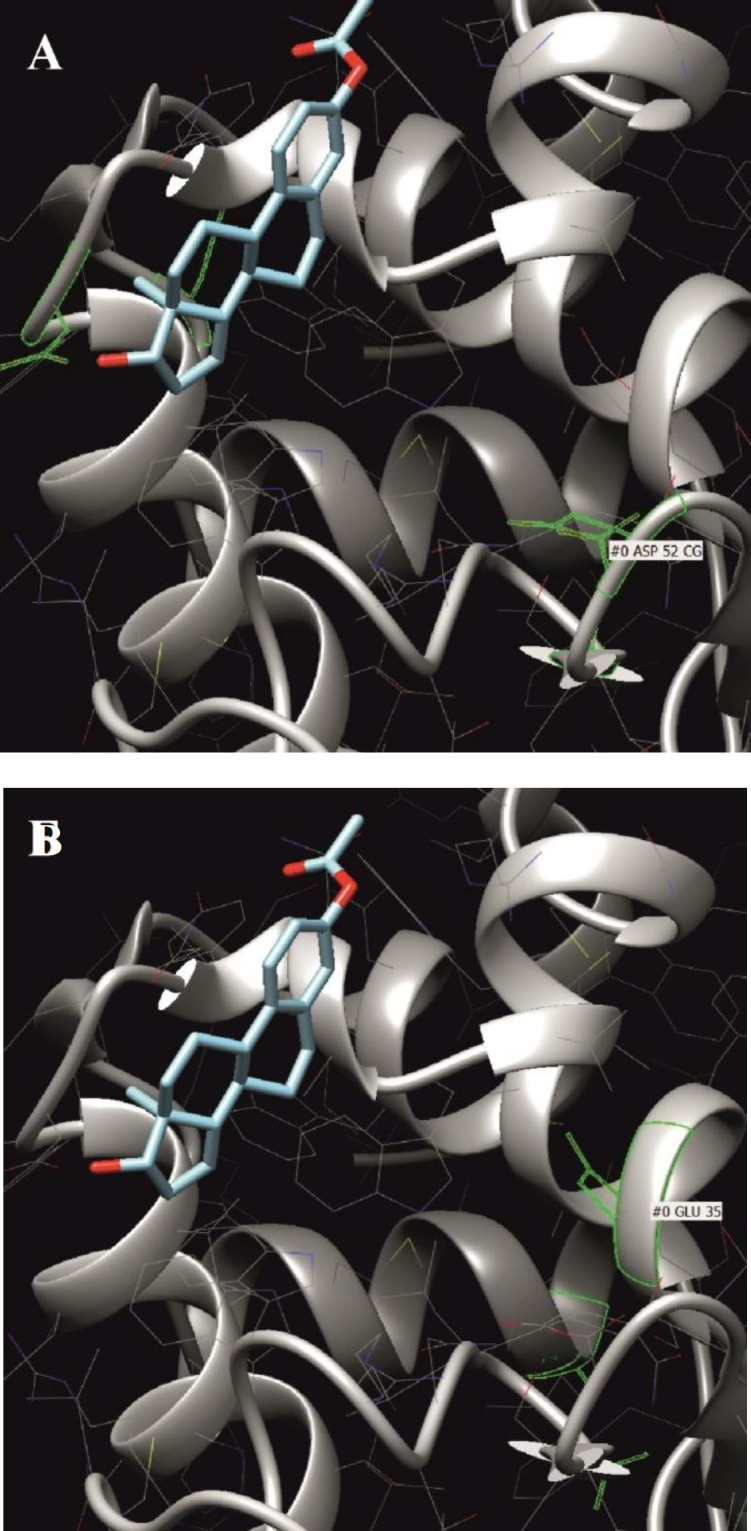
Position of the E2 molecule at the lowest Gibbs energy value in the lysozyme molecule. (A) relative to asp52 and (B) glu52. The asp52 and glu35 residues are highlighted in green color

In the range of ∆G values from -8.9149 to -8.9484, the EPN molecule was very close to both amino acids. At ∆G -9.1077, the 22^nd ^hydrogen atom of the ligand formed a hydrogen bond with asparagine at the 52^nd^ position, and its third hydrogen atom bound to glutamine at the 35^th^ position. Negative ∆G values confirmed that the reaction between proteins and catecholamines was spontaneous. When a number of combinations of spatial localization of hormone molecules are realized, and if the lysozyme molecule is attacked by several EPN molecules, the active site will be electrostatically altered, potentially affecting the enzymatic activity of the lysozyme molecule. It has been previously shown that the catecholamine precursor L-Dopa can bind to lysozyme and change its biophysical characteristics, such as its hydrodynamic radius and intrinsic fluorescence^[^^14^^]^. This situation is not the same for E2. Its activity against lysozyme is obviously not related to the attack of the active site but to a change in the conformation of the entire enzyme molecule. This observation was confirmed by the data of molecular docking, showing that the most energetically favorable associations of the ligand (E2) occur on the sections of the enzyme molecule remote from its active center (Fig. 4).

## DISCUSSION

Lysozyme, as a factor of animal innate immunity, is the most studied enzymes due to its importance in bacterial activity. Recently, its regulatory effect on gene transcription of the immune system of monocytes has been discovered^[^^15^^]^. Also, the conditions at which this enzyme has optimal inhibition activity, have been identified ^[^^7^^,^^16^^,^^17^^]^. However, much less is known about its mechanism of activation. It is believed that lysozyme is activated by positively charged molecules and inhibited by negatively charged ones^[^^18^^]^. Lysozyme is known to be activated by biotin^[^^19^^]^ and some hormones^[^^20^^]^. In this regard, Roberts et al.^[^^20^^]^ have found a significant increase in lysozyme activity under the influence of progesterone. In contrast, some studies have evaluated the effect of the stress hormone, cortisol, in the model organisms of channel catfish, and found no lysozyme activity following hormone treatment^[^^21^^]^. In some cases, hormones change the activity of enzymes depending on the type and concentration of the substrate. For instance, effect of EPN on the activity of pyruvate dehydrogenase in rat epididymal fat pads and their isolated adipocytes was evaluated following increasing the concentration of EPN, which in turn enhanced the enzyme activity^[^^22^^]^. A correlation between the activity of stress hormones (cortisol and EPN) and lysozyme in rainbow trout tissues has been reported upon exposure to hormone stress^[^^23^^]^. In our experiments, the impact of hormones and natriuretic peptides on the lysozyme molecule was direct, which allowed us to evaluate the degree of influence of these compounds on the acetyl muraminidase activity of enzyme.

**Table 2:** Changes in lysozyme activity following increasing concentrations of hormones at pH 6.3 and 5.5 at 34 °C compared to the control (%)

**Hormone/** **concentration (M)**	**рН** **6.3**	**рН** **5.5**
**EPN** 4.91 × 10^-11^ 24.55 × 10^-11^ 4.91 × 10^-10^	163 162 183	100 96 99
		
**NE** 3.55 × 10^-11^ 17.75 × 10^-11^ 3.55 × 10^-10^	96 100 107	105 100 101
		
**ANP** 6.49 × 10^-14^ 32.45 × 10^-14^ 6.49 × 10^-13^	116 127 149	112 115 121
		
**BNP** 7.22 × 10^-14^ 36.1 × 10^-14^ 7.22 × 10^-13^	88 86 99	104 108 108
		
**E** **2** 7.22 × 10^-14^ 36.1 × 10^-14^ 7.22 × 10^-13^	121 123 113	166 167 210

Under the impact of hormones, the values of lysozyme activity were widely varied relative to the control values. Nevertheless, the essential activation of lysozyme by EPN at temperatures of the skin surface at concentrations higher than the physiological ones suggests that in the stress situations, the enzyme shows more effective inhibitory properties against Gram-positive bacterial commensals of the skin^[^^8^^]^. Interestingly, despite the structural similarities of EPN and NE molecules, the latter behaved in exactly the opposite way. At temperatures of the skin, NE did not change lysozyme activity, while at 37 °C, the enzyme activity increased. NE is believed to be able to stimulate phagocytosis by neutrophiles^[^^24^^]^, which contain lysozyme granules. This process may explain the reason why neutrophils isolated from 24-hour wounds, unlike those from 120-hour wounds, demonstrated no NE-responsiveness of the phagocytic processes, which is in contrast to the circulating 24-hour and 120-hour neutrophils isolated from the restraint-stressed animals^[^^23^^]^. Nicholls et al.^[^^25^^]^ have demonstrated that NE increases the metabolic activity of neutrophiles proportionally to the incremental hormone concentration. In this study, myeloperoxidase concentration increased in the supernatants of the lysed neutrophiles treated with NE. Thus, we can assume that under the low temperature conditions, EPN activates that part of the lysozyme pool, which is localized on the skin. However, NE is more effective against the lysozyme synthesized by the cells of the immune system and in the tissues of internal organs. Although somewhat speculative, this hypothesis needs to be further tested in both *in vitro* and *in vivo* experiments, using model organisms. At 37 °C, the peptide factors ANP and BNP were found to stimulate lysozyme activity. 

The results of temperature activation of the mixture of E2 and lysozyme suggest the presence of both temperature- and pH-dependent natural mechanisms of the lysozyme activity regulated by this hormone *in vivo*. The optimal pH of human skin is known to be 4.7-5.5. In this context, it has been reported that the values between 4.5 and 4.7 are normal, based on the examination of the untreated skin^[^^26^^]^. Our study showed that when pH decreased to 5.5 at 34 °C, lysozyme activity returned to the control level under the influence of EPN and ANP. It has been recognized that the synthesis of both compounds is activated during physical activity accompanied by increased sweating and production of lysozyme. Under these conditions, the return of lysozyme activity to the level of the control values may be a preventive measure for protecting the useful skin commensals against the damaging effects of the enzyme.

Metabolically, NE is less active than EPN. At the same time, at physiological concentrations, the lysozyme activity slightly decreased at pH 5.5 compared to the background of the control. The muramidase activity of lysozyme and natriuretic peptide conjugates relatively differed from the control at pH 5.5, which is in agreement with their localization in plasma, where the pH is between 7.35-7.45, and it goes beyond the optimum activity of pure lysozyme (6.0-6.8). 

In the present study, the most interesting results obtained when analyzing the activity of a mixture of lysozyme and estrogen. Decreasing the pH to a value of 5.5, significantly increased its bacteriolytic activity in proportion to the concentration of the hormone. It has been reported that in healthy women, vaginal pH is regulated by the concentration of estrogen. In other words, increase in glycogen secretion due to estrogen stimulation, leads to the proliferation of lactobacilli and, consequently, acidification of the environment^[^^27^^]^. This process itself has a bacteriostatic effect on pathogenic microflora. Thus, increase in the lysozyme activity under the influence of estrogen in an acidic environment is an important factor contributes to the normalization of the composition of the vaginal microflora. Our findings have been confirmed by studies performed on the cervical fluid of women who took estrogen medications for a long time^[^^27^^]^. This study showed a significant increase in the lysozyme activity of intrauterine mucus against *Micrococcus lysodeikticus*. Ascomycetes, e.g. *Candida albicans*, play a huge role in the pathogenesis of the vagina, in addition to Gram-positive bacteria. The cell wall of these fungi includes a *β*-glucan-chitin cortex. Earlier, Krupyanskiiy *et al.*^[^^11^^]^ have reported the lysozyme activity against chitin. Thus, lysozyme is capable of restraining not only Gram-positive bacteria but also yeast fungi. 

Analysis of the ratio of the molar concentrations of lysozyme to hormones showed that there are 2.57-35.6 × 10^-6^ of catecholamine molecules and 1.6-16 × 10^-7^ of E2 molecules per enzyme molecule. This result indicates that the effect of hormones is not due to a change in the physicochemical environment, i.e., NaCl and Ca^2+[^^17^^] ^level. Indeed, it is mediated by binding to specific sites, and the nature of the reaction is spontaneous, verifying by the negative values of the Gibbs energy. 

As reported by Kaiser^[^^18^^]^, lysozyme is activated by positively charged catecholamine molecules. This activation was dependent on temperature and pH. In this regard, the activating role of EPN was pronounced at pH 6.3 and 34 °С and that of NE at 37 °С, but it did not depend on pH values. Recently, lysozyme has been recognized not only as a factor of nonspecific innate immunity but also as an enzyme controlling the bacterial population. It also has a number of immunomodulatory functions, including participation in inflammatory reactions^[^^28^^]^. 

In view of the fact that lysozyme-like enzymes participate in the processes of normal bacterial activity, its activation can lead to an imbalance in the development of both planktonic and biofilm forms of pathogenic bacteria. Our study demonstrates another possible way for regulating the lysozyme activity against human bacterial microflora through a mechanism of direct action of hormones on the reaction site of this enzyme molecule. The peculiarities of such regulation should be considered in clinical application for the increased doses of catecholamines and E2.

## DECLARATIONS

### Acknowledgments

We are grateful to Prof. V.K. Plakunov (S.N. Vinogradsky Institute of Microbiology, Russian Academy of Sciences Biotechnology Research Centre) for all the advice and comments in preparing the material for publication.

### Ethical statement

Not applicable.

### Data availability

The raw data supporting the conclusions of this article are available from the authors upon reasonable request.

### Author contributions

TP: conceptualized the study, obtained the experimental data, discussed the results, and wrote the manuscript; AG discussed the results, and wrote the manuscript; YN conceptualized the study, discussed the results, and wrote the manuscript.

### Conflict of interest

None declared.

### Funding/support

This work was carried out with funding from the Russian Science Foundation (grant 19-74-10071) and with partial support from the Ministry of Science and Higher Education, Russia.
